# Supplementary material for: Enhanced fusogenicity and pathogenicity of SARS-CoV-2 Delta P681R mutation
Source: Nature. 2021 Nov 25;602(7896):300–6. doi: 10.1038/s41586-021-04266-9 (PMC8828475; doi:10.1038/s41586-021-04266-9)
Supplement: Supplementary file 1 — Supplementary Fig. 1: gating strategy for flow cytometry of S protein expressing cells. Supplementary Fig. 2: Original (uncrossed) blots. [file 41586_2021_4266_MOESM1_ESM.pdf]

nature portfolio

https://doi.org/10.1038/s41586-021-04266-9

Supplementary information

Enhanced fusogenicity and pathogenicity of SARS-CoV-2 Delta P681R mutation

In the format provided by the authors and unedited

Supplementary Information

Enhanced fusogenicity and pathogenicity of SARS-CoV-2 Delta P681R mutation

Akatsuki Saito, Takashi Irie, Rigel Suzuki, Tadashi Maemura, Hesham Nasser, Keiya Uriu, Yusuke Kosugi, Kotaro Shirakawa, Kenji Sadamasu, Izumi Kimura, Jumpei Ito, Jiaqi Wu, Kiyoko Iwatsuki-Horimoto, Mutsumi Ito, Seiya Yamayoshi, Samantha Loeber, Masumi Tsuda, Lei Wang, Seiya Ozono, Erika P Butlertanaka, Yuri L Tanaka, Ryo Shimizu, Kenta Shimizu, Kumiko Yoshimatsu, Ryoko Kawabata, Takemasa Sakaguchi, Kenzo Tokunaga, Isao Yoshida, Hiroyuki Asakura, Mami Nagashima, Yasuhiro Kazuma, Ryosuke Nomura, Yoshihito Horisawa, Kazuhisa Yoshimura, Akifumi Takaori-Kondo, Masaki Imai, The Genotype to Phenotype Japan (G2P-Japan) Consortium, Shinya Tanaka, So Nakagawa, Terumasa Ikeda, Takasuke Fukuhara, Yoshihiro Kawaoka, Kei Sato

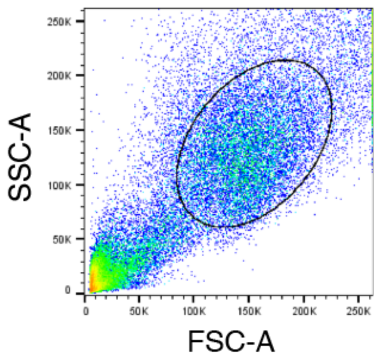

Supplementary Fig. 1. Gating strategy for flow cytometry of S protein-expressing cells.

A representative gating strategy for flow cytometry of S protein-expressing cells (Extended Data Fig. 8c) is shown.

Fig. 3d

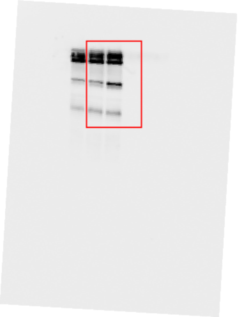

Mouse anti-SARS-CoV-2 S
monoclonal antibody
(clone 1A9, GeneTex,
Cat# GTX632604,
1:20,000)

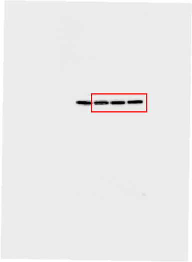

Rabbit anti-ACTB
monoclonal antibody
(clone 13E5, Cell Signalling,
Cat# 4970, 1:5,000)

Extended Data Fig. 14

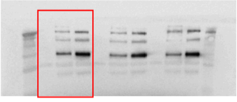

Mouse anti-SARS-CoV-2 S
monoclonal antibody
(clone 1A9, GeneTex,
Cat# GTX632604,
1:20,000)

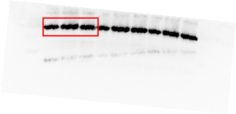

Mouse anti-HIV-1 p24
monoclonal antibody
(clone 183-H12-5C, obtained from
the HIV Reagent Program, NIH,
Cat# ARP-3537, 1:5,000)

Supplementary Fig. 2. Original (uncrossed) blots.

Uncrossed blots of Fig. 3d (left) and Extended Data Fig. 8a (right) are shown. Red boxes indicate the cropped area.
