# Supplementary material for: Enhanced fusogenicity and pathogenicity of SARS-CoV-2 Delta P681R mutation
Source: Nature. 2021 Nov 25;602(7896):300–6. doi: 10.1038/s41586-021-04266-9 (PMC8828475; doi:10.1038/s41586-021-04266-9)
Supplement: Supplementary file 2 — Reporting Summary [file 41586_2021_4266_MOESM2_ESM.pdf]

nature portfolio

Corresponding author(s): Tanaka, Nakagawa, Ikeda, Fukuhara, Kawaoka, Sato

Last updated by author(s): October 2, 2021

Reporting Summary

Nature Portfolio wishes to improve the reproducibility of the work that we publish. This form provides structure for consistency and transparency in reporting. For further information on Nature Portfolio policies, see our Editorial Policies and the Editorial Policy Checklist.

Statistics

For all statistical analyses, confirm that the following items are present in the figure legend, table legend, main text, or Methods section.

n/a	Confirmed	
☐	☒	The exact sample size (*n*) for each experimental group/condition, given as a discrete number and unit of measurement
☐	☒	A statement on whether measurements were taken from distinct samples or whether the same sample was measured repeatedly
☐	☒	The statistical test(s) used AND whether they are one- or two-sided<br>*Only common tests should be described solely by name; describe more complex techniques in the Methods section.*
☒	☐	A description of all covariates tested
☒	☐	A description of any assumptions or corrections, such as tests of normality and adjustment for multiple comparisons
☐	☒	A full description of the statistical parameters including central tendency (e.g. means) or other basic estimates (e.g. regression coefficient) AND variation (e.g. standard deviation) or associated estimates of uncertainty (e.g. confidence intervals)
☐	☒	For null hypothesis testing, the test statistic (e.g. *F*, *t*, *r*) with confidence intervals, effect sizes, degrees of freedom and *P* value noted<br>*Give P values as exact values whenever suitable.*
☒	☐	For Bayesian analysis, information on the choice of priors and Markov chain Monte Carlo settings
☒	☐	For hierarchical and complex designs, identification of the appropriate level for tests and full reporting of outcomes
☒	☐	Estimates of effect sizes (e.g. Cohen's *d*, Pearson's *r*), indicating how they were calculated

Our web collection on statistics for biologists contains articles on many of the points above.

Software and code

Policy information about availability of computer code

Data collection	MAFFT suite v7.407, IQ-TREE v2.1.3, FinePointe software v2.8.0.12146 (Data Sciences International), CosmoScan Database software v3.3.27.100 (Rigaku Corporation)
Data analysis	BZ-X800 Analyzer v1.1.2.4 (Keyence), Image Studio Lite v5.2 (LI-COR Biosciences), Fiji v2.2.0 in ImageJ v2.2.0, CosmoScan Database software v3.3.27.100 (Rigaku Corporation), Sequencher software v5.1 (Gene Codes Corporation), Excel software v16.16.8 (Microsoft), Photoshop 2020 v21.0.2 (Adobe)

For manuscripts utilizing custom algorithms or software that are central to the research but not yet described in published literature, software must be made available to editors and reviewers. We strongly encourage code deposition in a community repository (e.g. GitHub). See the Nature Portfolio guidelines for submitting code & software for further information.

Data

Policy information about availability of data

All manuscripts must include a data availability statement. This statement should provide the following information, where applicable:

- Accession codes, unique identifiers, or web links for publicly available datasets
- A description of any restrictions on data availability
- For clinical datasets or third party data, please ensure that the statement adheres to our policy

The raw data of virus sequences analysed in this study are deposited in Gene Expression Omnibus (accession number: GSE182738). Publicly available viral sequence data are available from GISAID database. The accession numbers of viral sequences used in this study are listed in Method section.

Field-specific reporting

Please select the one below that is the best fit for your research. If you are not sure, read the appropriate sections before making your selection.

- [x] Life sciences
- [ ] Behavioural & social sciences
- [ ] Ecological, evolutionary & environmental sciences

For a reference copy of the document with all sections, see nature.com/documents/nr-reporting-summary-flat.pdf

Life sciences study design

All studies must disclose on these points even when the disclosure is negative.

Sample size	The sample sizes (n > 3) for cell culture experiments were chosen for applying statistical tests. The sample sizes (n > 4) for the hamster studies were chosen because they have previously been shown to be sufficient to evaluate a significant difference among groups (Belser et al., Nature, 2013; Zhang et al., Science, 2013; Imai et al., Nature Microbiology, 2020).
Data exclusions	No data were excluded.
Replication	In vitro experiments representative of at least 2 experiments with multiple samples per time point. In vivo experiments (hamster) utilized multiple animals per group per time point and were from more than single experiment. In vivo experiments were replicated and performed independently. All attempts at replication were successful.
Randomization	No method of randomization was used to determine how the animals were allocated to the experimental groups and processed in this study, because covariates (sex and age) were identical (male, 4 weeks old). For experiments other than animal studies, randomization is not applicable because homogenous materials (i.e., cell lines) were used. Primary human nasal epithelial cells were used in an experiment, but only one donor was used. Therefore, randomization is not applicable.
Blinding	For the microCT imaging (Extended Data Fig. 19), images were anonymized and randomized; the scorer was blinded to the group allocation. No blinding was carried out for the other experiments, because these are not relevant for an observational study.

Reporting for specific materials, systems and methods

We require information from authors about some types of materials, experimental systems and methods used in many studies. Here, indicate whether each material, system or method listed is relevant to your study. If you are not sure if a list item applies to your research, read the appropriate section before selecting a response.

Materials & experimental systems

n/a	Involved in the study	
[ ]	[x]	Antibodies
[ ]	[x]	Eukaryotic cell lines
[x]	[ ]	Palaeontology and archaeology
[ ]	[x]	Animals and other organisms
[ ]	[x]	Human research participants
[x]	[ ]	Clinical data
[x]	[ ]	Dual use research of concern

Methods

n/a	Involved in the study	
[x]	[ ]	ChIP-seq
[ ]	[x]	Flow cytometry
[x]	[ ]	MRI-based neuroimaging

Antibodies

Antibodies used	For Western blotting:<br>Mouse anti-SARS-CoV-2 S monoclonal antibody (clone 1A9, GeneTex, Cat# GTX632604, 1:20,000)<br>Rabbit anti-ACTB monoclonal antibody (clone 13E5, Cell Signalling, Cat# 4970, 1:5,000)<br>Mouse anti-HIV-1 p24 monoclonal antibody (clone 183-H12-5C, obtained from the HIV Reagent Program, NIH, Cat# ARP-3537, 1:5,000)<br>Horseradish peroxidase (HRP)-conjugated donkey anti-rabbit IgG polyclonal antibody (Jackson ImmunoResearch, Cat# 711-035-152, 1:10,000)<br>HRP-conjugated donkey anti-mouse IgG polyclonal antibody (Jackson ImmunoResearch, Cat# 715-035-150, 1:10,000).<br><br>For flow cytometry:<br>Rabbit anti-SARS-CoV-2 S monoclonal antibody (clone HL6, GeneTex, Cat# GTX635654, 1:200)<br>Rabbit anti-SARS-CoV-2 S S1/S2 polyclonal antibody (Thermo Fisher Scientific, Cat# PA5-112048, 1:100)<br>APC-conjugated goat anti-rabbit IgG polyclonal antibody (Jackson ImmunoResearch, Cat# 111-136-144, 1:100)<br><br>Normal rabbit IgG (SouthernBiotech, Cat# 0111-01, 1:100)

For immunohistolochemistry:
Mouse anti-SARS-CoV-2 N monoclonal antibody (clone 1035111, R&D systems, Cat# MAB10474-SP, 1:400)

For neutralisation assay:
Human anti-SARS-CoV-2 RBD neutralizing antibodies (clone 8A5, Elabscience, Cat# E-AB-V1021; clone 4A3, Elabscience, Cat# E-AB-V1024; and clone CB6, Elabscience, Cat# E-AB-V1028)

Validation	Validation was conducted by manufacturers prior to sale, and validation statements are available on the manufacturers' website.

Eukaryotic cell lines

Policy information about cell lines

Cell line source(s)	HEK293 cells (a human embryonic kidney cell line; ATCC CRL-1573)<br>HEK293T cells (a human embryonic kidney cell line; ATCC CRL-3216)<br>HOS cells (a human osteosarcoma cell line; ATCC CRL-1543)<br>Vero cells [an African green monkey (Chlorocebus sabaeus) kidney cell line; JCRB0111]<br>VeroE6/TMPRSS2 cells [an African green monkey (Chlorocebus sabaeus) kidney cell line; JCRB1819]<br>Calu-3 cells (a human lung epithelial cell line; ATCC HTB-55)<br>HEK293-C34 cells (Torii et al., Cell Reports, 2020)<br>HOS-ACE2/TMPRSS2 cells: prepared as previously described (Ferreira et al., bioRxiv, 2021; Ozono et al., Nat Commun, 2021).<br>Primary human nasal epithelial cells: purchased from Epithelix (Cat# EP01, Batch# MD0436).
Authentication	None of the cells used were authenticated.
Mycoplasma contamination	All cell lines were regularly tested for mycoplasma contamination by using PCR and were confirmed to be mycoplasma-free.
Commonly misidentified lines<br>(See ICLAC register)	No commonly misidentified cell lines were used.

Animals and other organisms

Policy information about studies involving animals; ARRIVE guidelines recommended for reporting animal research

Laboratory animals	Syrian hamsters (male, 4 weeks old) were purchased from Japan SLC Inc. (Shizuoka, Japan).
Wild animals	No wild animal was used in this study.
Field-collected samples	No field collected sample was used in the study.
Ethics oversight	All experiments with hamsters were performed in accordance with the Science Council of Japan’s Guidelines for Proper Conduct of Animal Experiments. The protocols were approved by the Institutional Animal Care and Use Committee of National University Corporation Hokkaido University (approval number 20-0123) and the Animal Experiment Committee of the Institute of Medical Science, the University of Tokyo (approval number PA19-75).

Note that full information on the approval of the study protocol must also be provided in the manuscript.

Human research participants

Policy information about studies involving human research participants

Population characteristics	Peripheral blood was collected four weeks after the second vaccination with BNT162b2 (Pfizer-BioNTech), and sera were isolated from the peripheral blood of 19 vaccinees (average age: 38, range: 28-59, 26% male).
Recruitment	The voluntary donors were the Kyoto University Hospital staffs four weeks after the second vaccination with BNT162b2. The donors were recruited by massive mail invitation regardless of age, sex, gender, race, ethnicity, or other characteristics. Written informed consent was obtained from the voluntary donor. The sera used in this study were selected randomly.
Ethics oversight	All protocols involving specimens from human subjects recruited at Kyoto University were reviewed and approved by the Institutional Review Boards of Kyoto University (approval number G0697) and the Institute of Medical Science, the University of Tokyo (approval number 2021-1-0416).

Note that full information on the approval of the study protocol must also be provided in the manuscript.

Flow Cytometry

Plots

Confirm that:

- [x] The axis labels state the marker and fluorochrome used (e.g. CD4-FITC).
- [x] The axis scales are clearly visible. Include numbers along axes only for bottom left plot of group (a 'group' is an analysis of identical markers).
- [x] All plots are contour plots with outliers or pseudocolor plots.
- [x] A numerical value for number of cells or percentage (with statistics) is provided.

Methodology

Sample preparation	HEK293 cells were cotransfected with 400 ng of D614G S or D614G/P681R expression plasmids and 400 ng pDSP1-7 using TransIT-LT1 (Takara, Cat# MIR2300).
Instrument	FACS Canto II instrument (BD Biosciences)
Software	FlowJo
Cell population abundance	10,000 cells gated in the FSC-A/SSC-A plot (Extended Data Fig. 15a) were acquired for each condition.
Gating strategy	Shown in Supplementary Fig. 1.

- [x] Tick this box to confirm that a figure exemplifying the gating strategy is provided in the Supplementary Information.
